# The genome sequence of the dumpy grass hoverfly, *Melanostoma mellinum *(Linnaeus, 1758)

**DOI:** 10.12688/wellcomeopenres.17615.1

**Published:** 2022-02-15

**Authors:** William Hawkes, Karl Wotton, University of Oxford and Wytham Woods Genome Acquisition Lab, Darwin Tree of Life Barcoding collective, Wellcome Sanger Institute Tree of Life programme, Wellcome Sanger Institute Scientific Operations: DNA Pipelines collective, Tree of Life Core Informatics collective, Darwin Tree of Life Consortium

**Affiliations:** 1 Centre for Ecology and Conservation, Department of Biosciences, University of Exeter, Penryn, UK

**Keywords:** Melanostoma mellinum, dumpy grass hoverfly, genome sequence, chromosomal, Diptera

## Abstract

We present a genome assembly from an individual male *Melanostoma mellinum *(the dumpy grass hoverfly; Arthropoda; Insecta; Diptera; Syriphidae). The genome sequence is 731 megabases in span. The majority of the assembly (99.67%) is scaffolded into five chromosomal pseudomolecules, with the X and Y sex chromosomes assembled. The complete mitochondrial genome was also assembled and is 16.1 kilobases in length.

## Species taxonomy

Eukaryota; Metazoa; Ecdysozoa; Arthropoda; Hexapoda; Insecta; Pterygota; Neoptera; Endopterygota; Diptera; Brachycera; Muscomorpha; Syrphoidea; Syrphidae; Syrphinae; Melanostomini; Melanostoma; *Melanostoma mellinum* (Linnaeus, 1758) (NCBI:txid653684).

## Background

**Figure 1.**
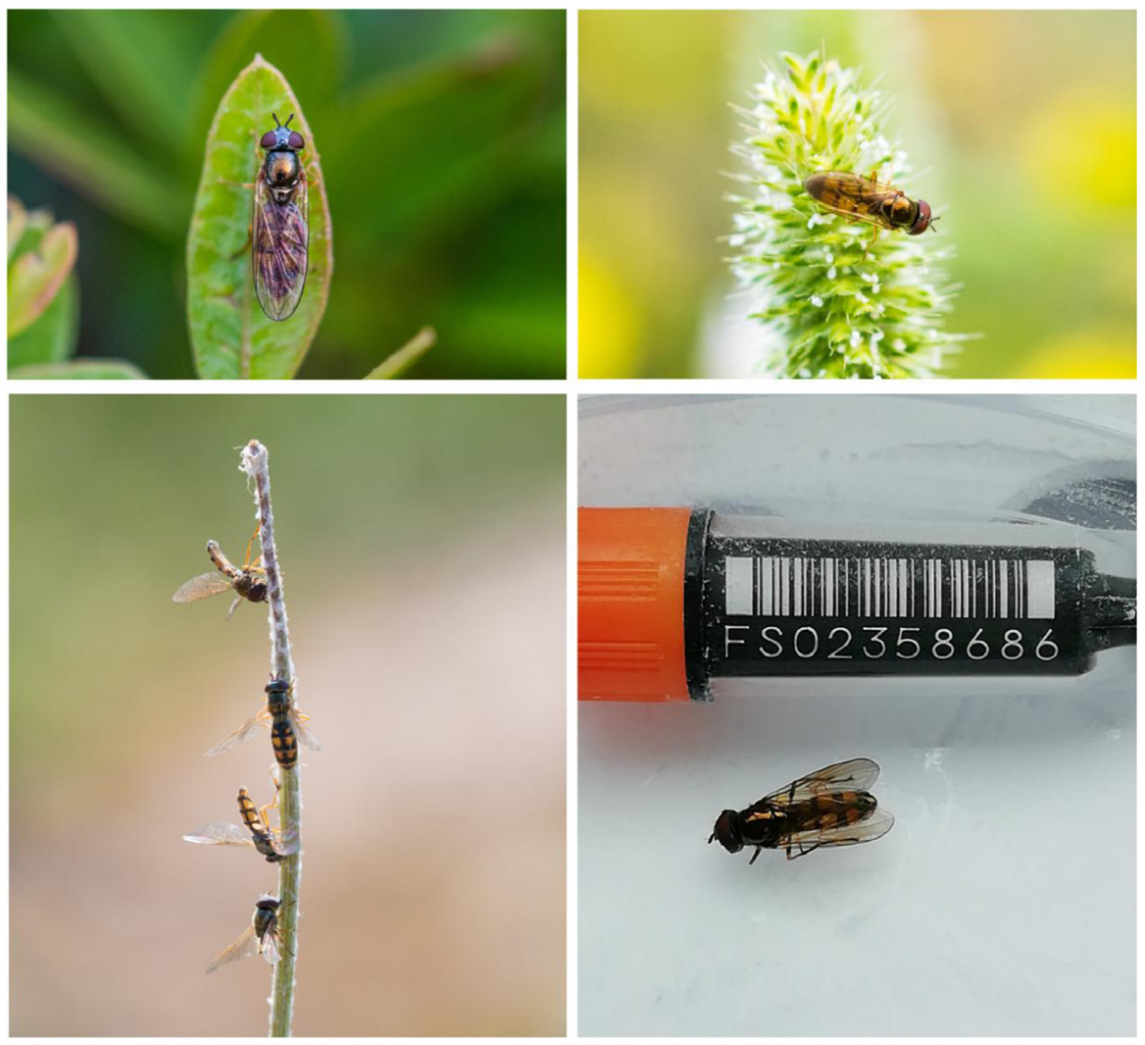
Example images of *Melanostoma mellinum*. Top: female (left) and male (right) examples of *M. mellinum*; photographs provided by WH. Bottom left: examples of dead *M. mellinum* individuals that have succumbed to the *Entomophora* fungal pathogen; photograph provided by WH. Bottom right: the male *M. mellinum* specimen used for genome sequencing; photograph provided by Liam Crowley, University of Oxford.

*Melanostoma mellinum* is a small hoverfly with yellow and black markings, perhaps using this aposematic colourway to loosely mimic wasps and so gain protection from predators ( Ball & Morris, 2015; van Veen, 2010). These hoverflies have dark faces and dark scutellums. Female *Melanostoma* species have distinctive inverted yellow triangle abdominal markings and can be separated from the other common species *M. scalare* by the narrow dust spots on the otherwise shining frons ( Ball & Morris, 2015). Male *M. mellinum* ( Figure 1) have square yellow abdominal markings with tergite 2 and 3 being as long as wide, while *M. scalare* males have longer tergites ( Ball & Morris, 2015; van Veen, 2010). Colour and morphological variation is high and *M. mellinum* may comprise a species complex ( Haarto & Ståhls, 2014). *M. mellinum* is an abundant grassland species in the UK and can be found in greatest numbers during summer, feeding on the pollen of wind pollinated flowers such as grasses, plantain and sedges ( Ball & Morris, 2015). The larvae of this species feed on aphids in the leaf litter and ground layer and are incredibly generalist predators, preying on at least 32 different aphid species ( Dziock, 2005) and gall forming Psyllidae ( Hodkinson & Flint, 1971). Like some other generalist hoverflies, *M. mellinum* adults display seasonal migratory behaviour ( Aubert *et al*., 1976; Gatter & Schmid, 1990). This species seems to be heavily affected by the *Entomophora* fungal pathogen ( Jensen & Eilenberg, 2001) and dead *M. mellinum* individuals can be often found hanging beneath flower heads with fungal spores exuding (WH, personal observation; Figure 1). Migration in this species may function to reduce the negative effects of this fungus on the population by allowing escape from contaminated habitats and by culling infected animals, both phenomena seen in other migrants (see Satterfield *et al*. (2018)). This is the first production of a high quality *M. mellinum* genome and we believe that the sequence described here, generated as part of the Darwin Tree of Life project, will further aid understanding of the biology and ecology of this hoverfly.

## Genome sequence report

The genome was sequenced from a single male *M. mellinum* collected from Wytham Great Wood, Oxfordshire (Biological vice-county: Berkshire), UK (latitude 51.769, longitude -1.330) ( Figure 1). A total of 26-fold coverage in Pacific Biosciences single-molecule long reads and 55-fold coverage in 10X Genomics read clouds were generated. Primary assembly contigs were scaffolded with chromosome conformation Hi-C data. Manual assembly curation corrected 332 missing/misjoins and removed 35 haplotypic duplications, reducing the assembly size by 2.84% and the scaffold number by 74.75%, and increasing the scaffold N50 by 395.90%.

**Figure 2.**
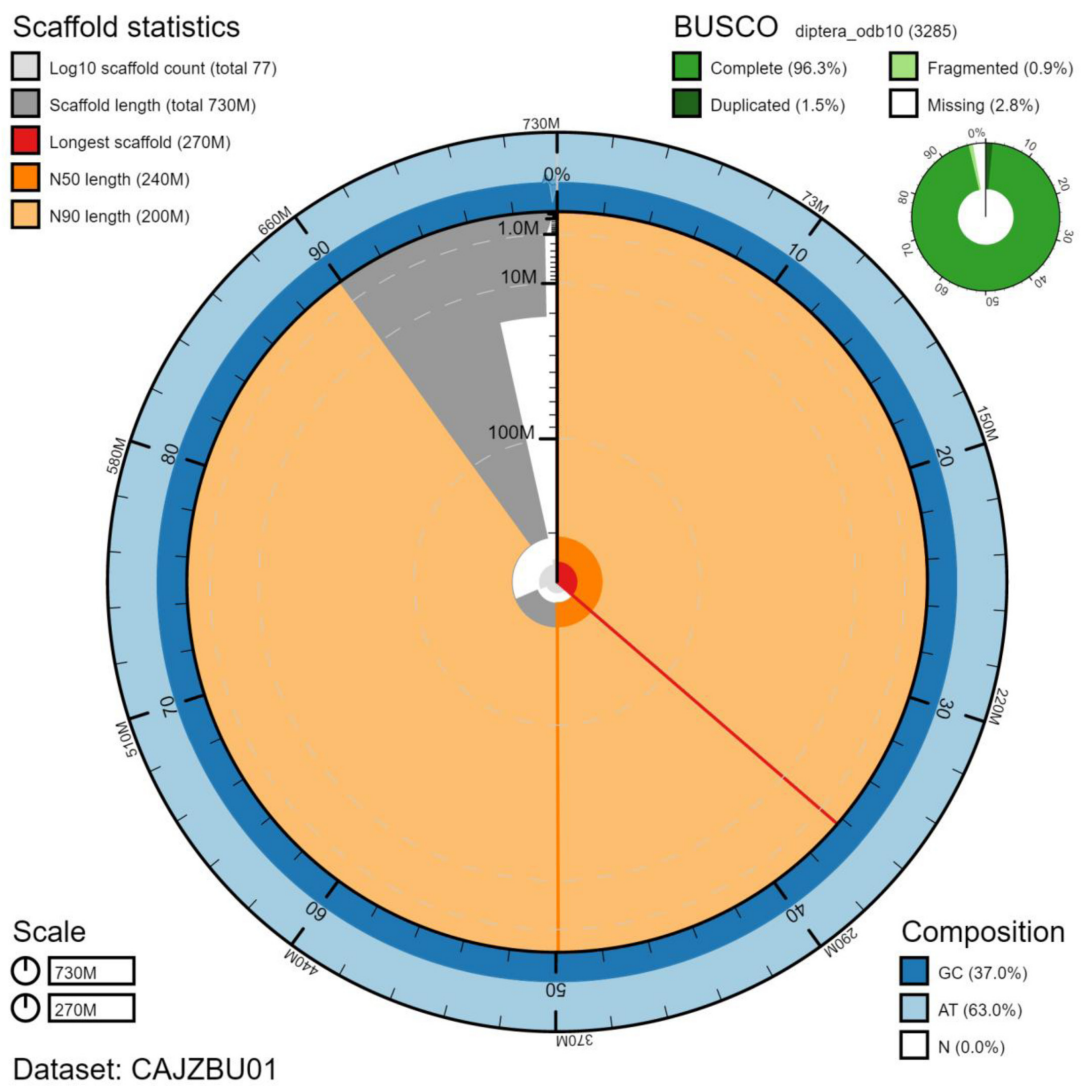
Genome assembly of *Melanostoma mellinum*, idMelMell2.1: metrics. The BlobToolKit Snailplot shows N50 metrics and BUSCO gene completeness. The main plot is divided into 1,000 size-ordered bins around the circumference with each bin representing 0.1% of the 731,040,377 bp assembly. The distribution of scaffold lengths is shown in dark grey with the plot radius scaled to the longest scaffold present in the assembly (265,601,162 bp, shown in red). Orange and pale-orange arcs show the N50 and N90 scaffold lengths (235,131,548 and 204,347,527 bp), respectively. The pale grey spiral shows the cumulative scaffold count on a log scale with white scale lines showing successive orders of magnitude. The blue and pale-blue area around the outside of the plot shows the distribution of GC, AT and N percentages in the same bins as the inner plot. A summary of complete, fragmented, duplicated and missing BUSCO genes in the diptera_odb10 set is shown in the top right. An interactive version of this figure is available at https://blobtoolkit.genomehubs.org/view/idMelMell2.1/dataset/CAJZBU01/snail.

The final assembly has a total length of 731 Mb in 76 sequence scaffolds with a scaffold N50 of 235 Mb ( Table 1). The majority, 99.67%, of the assembly sequence was assigned to five chromosomal-level scaffolds, representing three autosomes (numbered by sequence length), and the X and Y sex chromosomes ( Figure 2– Figure 5; Table 2). The assembly has a BUSCO v5.1.2 ( Simão *et al*., 2015) completeness of 96.3% (single 94.8%, duplicated 1.5%) using the diptera_odb10 reference set (n=3285). While not fully phased, the assembly deposited is of one haplotype. Contigs corresponding to the second haplotype have also been deposited.

**Table 1.** Genome data for *Melanostoma mellinum*, idMelMell2.1.

*Project accession data*	
Assembly identifier	idMelMell2.1
Species	*Melanostoma mellinum*
Specimen	idMelMell2
NCBI taxonomy ID	653684
BioProject	PRJEB46300
BioSample ID	SAMEA7520051
Isolate information	Male, head/thorax (genome assembly), abdomen (Hi-C)
*Raw data accessions*	
PacificBiosciences SEQUEL II	ERR6636092, ERR6636093
10X Genomics Illumina	ERR6688425-ERR6688428
Hi-C Illumina	ERR6688429
*Genome assembly*	
Assembly accession	GCA_914767635.1
*Accession of alternate haplotype*	GCA_914767615.1
Span (Mb)	731
Number of contigs	479
Contig N50 length (Mb)	4.6
Number of scaffolds	76
Scaffold N50 length (Mb)	235
Longest scaffold (Mb)	266
BUSCO * genome score	C:96.3%[S:94.8%,D:1.5%],F:0.9%,M:2.8%,n:3285

*BUSCO scores based on the diptera_odb10 BUSCO set using v5.1.2. C= complete [S= single copy, D=duplicated], F=fragmented, M=missing, n=number of orthologues in comparison. A full set of BUSCO scores is available at https://blobtoolkit.genomehubs.org/view/idMelMell2.1/dataset/CAJZBU01/busco.

**Figure 3.**
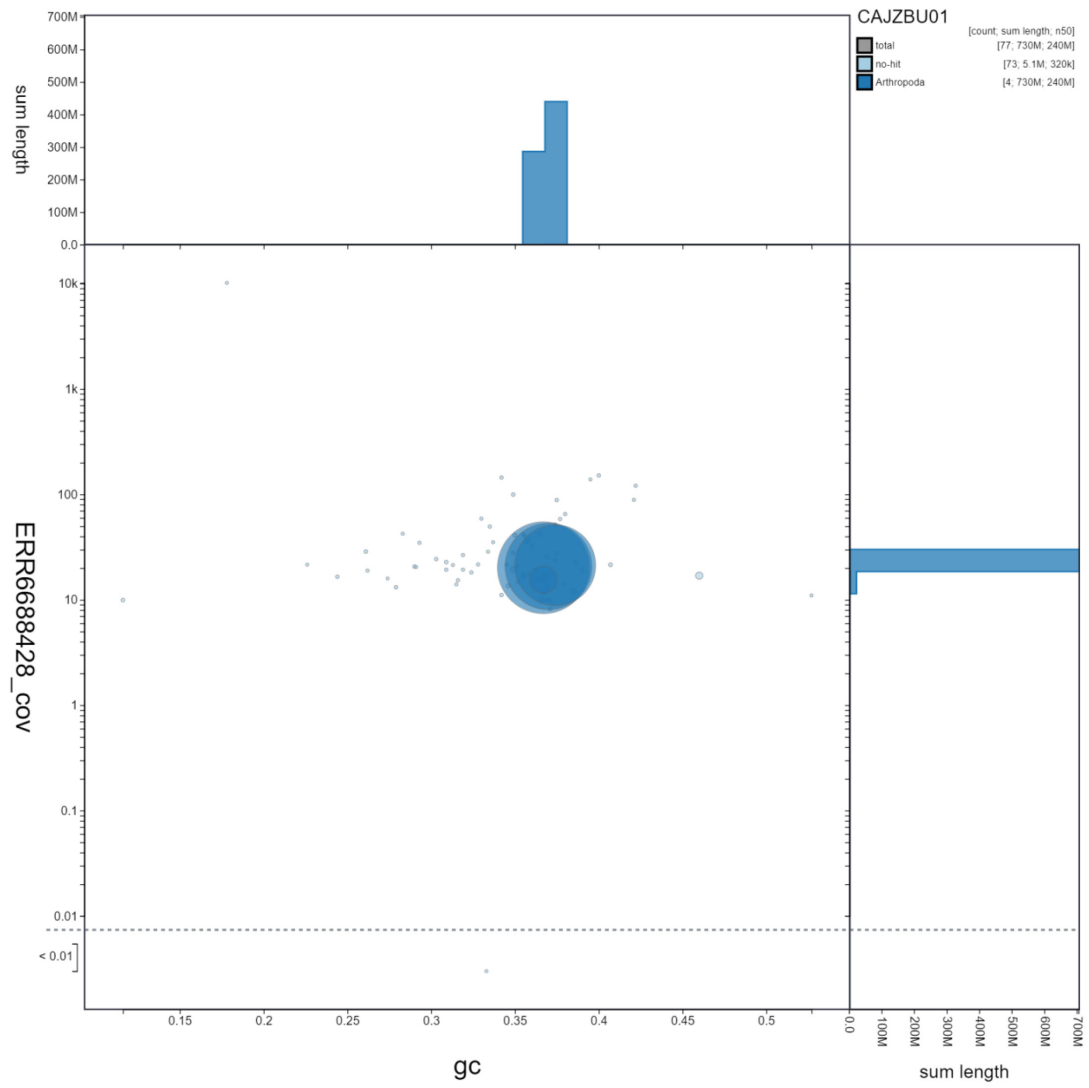
Genome assembly of *Melanostoma mellinum*, idMelMell2.1: GC coverage. BlobToolKit GC-coverage plot. Scaffolds are coloured by phylum. Circles are sized in proportion to scaffold length. Histograms show the distribution of scaffold length sum along each axis. An interactive version of this figure is available at https://blobtoolkit.genomehubs.org/view/idMelMell2.1/dataset/CAJZBU01/blob.

**Figure 4.**
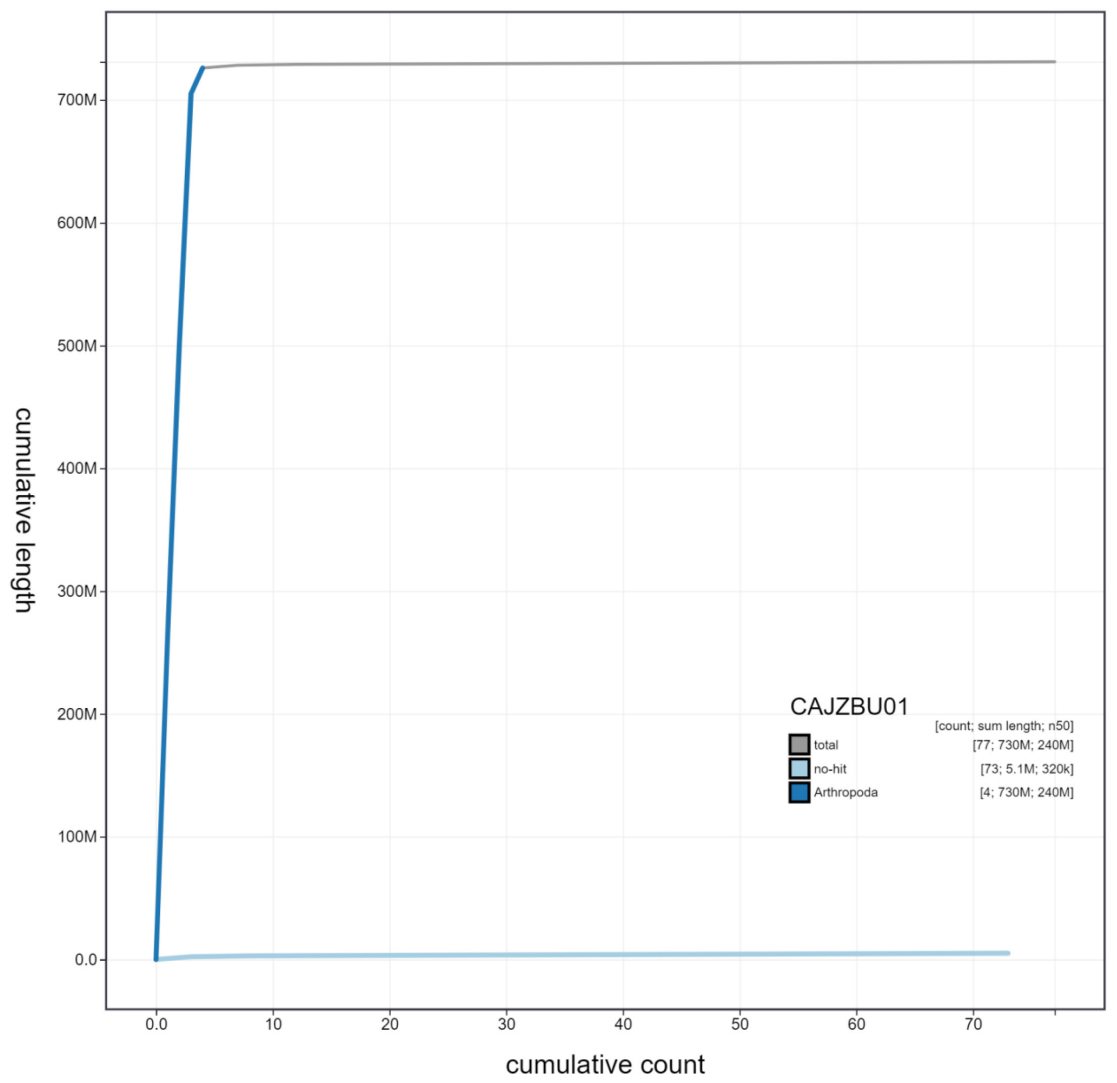
Genome assembly of *Melanostoma mellinum*, idMelMell2.1: cumulative sequence. BlobToolKit cumulative sequence plot. The grey line shows cumulative length for all scaffolds. Coloured lines show cumulative lengths of scaffolds assigned to each phylum using the buscogenes taxrule. An interactive version of this figure is available at https://blobtoolkit.genomehubs.org/view/idMelMell2.1/dataset/CAJZBU01/cumulative.

**Figure 5.**
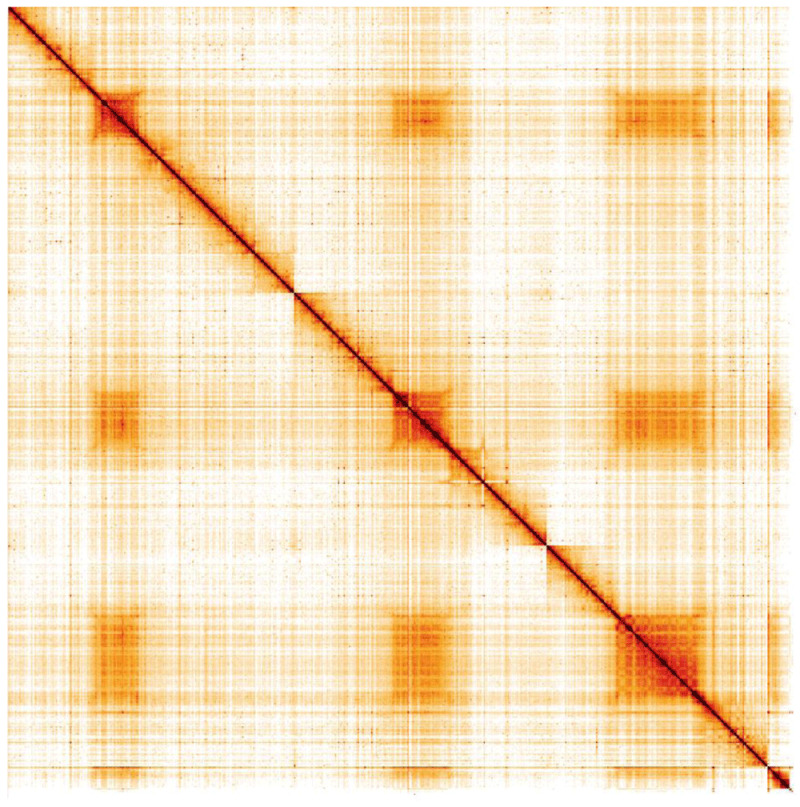
Genome assembly of *Melanostoma mellinum*, idMelMell2.1: Hi-C contact map. Hi-C contact map of the idMelMell2.1 assembly, visualised in HiGlass. Chromosomes are arranged in size order from left to right and top to bottom.

**Table 2.** Chromosomal pseudomolecules in the genome assembly of *Melanostoma mellinum*, idMelMell2.1.

INSDC accession	Chromosome	Size (Mb)	GC%
OU612058.1	1	265.60	36.7
OU612059.1	2	235.13	37.0
OU612060.1	3	204.35	37.4
OU612061.1	X	20.89	36.7
OU612062.1	Y	1.11	36.8
OU612063.1	MT	0.02	18.7
-	Unplaced	3.94	37.0

## Methods

### Sample acquisition, DNA extraction and sequencing

One male *M. mellinum* sample, idMelMell2, was collected from Wytham Great Wood, Oxfordshire, (Biological vice-county: Berkshire), UK (latitude 51.769, longitude -1.330) by Will Hawkes, University of Exeter on 7 August 2019. The specimen was caught with a net, identified by the same individual, snap-frozen on dry ice and stored using a CoolRack.

DNA was extracted from the head/thorax of idMelMell2 at the Wellcome Sanger Institute (WSI) Scientific Operations core from the whole organism using the Qiagen MagAttract HMW DNA kit, according to the manufacturer’s instructions. Pacific Biosciences HiFi circular consensus and 10X Genomics Chromium read cloud sequencing libraries were constructed according to the manufacturers’ instructions. Sequencing was performed by the Scientific Operations core at the Wellcome Sanger Institute on Pacific Biosciences SEQUEL II (HiFi) and Illumina HiSeq X (10X) instruments. Hi-C data were generated from abdomen tissue of idMelMell2 using the Arima v2 Hi-C kit in the Tree of Life laboratory and sequenced at the Scientific Operations core on an Illumina NovaSeq 6000 instrument.

### Genome assembly

Assembly was carried out with Hifiasm ( Cheng *et al*., 2021); haplotypic duplication was identified and removed with purge_dups ( Guan *et al*., 2020). One round of polishing was performed by aligning 10X Genomics read data to the assembly with longranger align, calling variants with freebayes ( Garrison & Marth, 2012). The assembly was then scaffolded with Hi-C data ( Rao *et al*., 2014) using SALSA2 ( Ghurye *et al*., 2019). The assembly was checked for contamination as described previously ( Howe *et al*., 2021). Manual curation was performed using HiGlass ( Kerpedjiev *et al*., 2018) and Pretext. The mitochondrial genome was assembled using MitoHiFi ( Uliano-Silva *et al*., 2021), which performs annotation using MitoFinder ( Allio *et al*., 2020). The genome was analysed and BUSCO scores generated within the BlobToolKit environment ( Challis *et al*., 2020). Table 3 contains a list of all software tool versions used, where appropriate.

**Table 3.** Software tools used.

Software tool	Version	Source
Hifiasm	0.15.2	Cheng *et al*., 2021
purge_dups	1.2.3	Guan *et al*., 2020
SALSA2	2.2	Ghurye *et al*., 2019
longranger align	2.2.2	https://support.10xgenomics.com/genome-exome/ software/pipelines/latest/advanced/other-pipelines
freebayes	1.3.1-17-gaa2ace8	Garrison & Marth, 2012
MitoHiFi	2.0	Uliano-Silva *et al*., 2021
HiGlass	1.11.6	Kerpedjiev *et al*., 2018
PretextView	0.2.x	https://github.com/wtsi-hpag/PretextView
BlobToolKit	2.6.4	Challis *et al*., 2020

### Ethics/compliance issues

The materials that have contributed to this genome note have been supplied by a Darwin Tree of Life Partner. The submission of materials by a Darwin Tree of Life Partner is subject to the Darwin Tree of Life Project Sampling Code of Practice. By agreeing with and signing up to the Sampling Code of Practice, the Darwin Tree of Life Partner agrees they will meet the legal and ethical requirements and standards set out within this document in respect of all samples acquired for, and supplied to, the Darwin Tree of Life Project. Each transfer of samples is further undertaken according to a Research Collaboration Agreement or Material Transfer Agreement entered into by the Darwin Tree of Life Partner, Genome Research Limited (operating as the Wellcome Sanger Institute), and in some circumstances other Darwin Tree of Life collaborators.

## Data availability

European Nucleotide Archive: Melanostoma mellinum (dumpy grass hoverfly). Accession number PRJEB46300; https://identifiers.org/ena.embl/PRJEB46300.

The genome sequence is released openly for reuse. The *M. mellinum* genome sequencing initiative is part of the Darwin Tree of Life (DToL) project. All raw sequence data and the assembly have been deposited in INSDC databases. The genome will be annotated and presented through the Ensembl pipeline at the European Bioinformatics Institute. Raw data and assembly accession identifiers are reported in Table 1.

## Author information

Members of the University of Oxford and Wytham Woods Genome Acquisition Lab are listed here: https://doi.org/10.5281/zenodo.5746938.

Members of the Darwin Tree of Life Barcoding collective are listed here: https://doi.org/10.5281/zenodo.5744972.

Members of the Wellcome Sanger Institute Tree of Life programme are listed here: https://doi.org/10.5281/zenodo.5744840.

Members of Wellcome Sanger Institute Scientific Operations: DNA Pipelines collective are listed here: https://doi.org/10.5281/zenodo.5746904.

Members of the Tree of Life Core Informatics collective are listed here: https://doi.org/10.5281/zenodo.5743293.

Members of the Darwin Tree of Life Consortium are listed here: https://doi.org/10.5281/zenodo.5638618.
