# Supplementary material for: The genome sequence of the dumpy grass hoverfly, Melanostoma mellinum (Linnaeus, 1758)
Source: Wellcome Open Res. 2022 Feb 15;7:59. [Version 1] doi: 10.12688/wellcomeopenres.17615.1 (PMC9975431; doi:10.12688/wellcomeopenres.17615.1)
Supplement: Peer review history

# Peer review: Reviewer response for version 1

**DOI:** 10.21956/wellcomeopenres.19486.r53457

**Published:** 2022-12-12

**Authors:** Renata Schama

**Affiliations:** 1 Instituto Nacional de Ciência e Tecnologia em Entomologia Molecular-INCT-EM, Rio de Janeiro, Brazil; 2 Fiocruz, Rio de Janeiro, Brazil

The Data Note describes how the genome sequence of the dumpy grass hoverfly, Melanostoma millenum, was sequenced. It clearly describes all the methodologies used, from collection, DNA extraction, DNA sequencing, and computational methods. Although they did DNA extraction of a whole body part the sequencing technology and posterior analyses were optimal in cleaning up the data and making sure they could build the different chromosomes. All quality metrics were good and the final assembly is well-supported with high completeness. The data is readily accessible, making sure the computational genomics part of the work could be replicated and the final result is freely available for use. Overall this is a clear and well-performed work.

Are sufficient details of methods and materials provided to allow replication by others?

Yes

Is the rationale for creating the dataset(s) clearly described?

Yes

Are the datasets clearly presented in a useable and accessible format?

Yes

Are the protocols appropriate and is the work technically sound?

Yes

Reviewer Expertise:

Population genetics, and gene family evolution with a focus on arthropod genomes (particularly mosquitoes).

I confirm that I have read this submission and believe that I have an appropriate level of expertise to confirm that it is of an acceptable scientific standard.

# Peer review: Reviewer response for version 1

**DOI:** 10.21956/wellcomeopenres.19486.r48743

**Published:** 2022-02-16

**Authors:** Thomas Pauli

**Affiliations:** 1 Institute of Medical Bioinformatics and Systems Medicine, Faculty of Medicine, Medical Center-University of Freiburg, University of Freiburg, Freiburg, Germany

The data note article written by Hawkes and Wotton describes the sequencing and subsequent assembly of the *Melanostoma mellinum *genome using long-read sequencing on a PacBio platform.

 In my estimation, the authors succeeded in generating a well-documented and accessible dataset. I only have minor criticism regarding the data note’s writing style and presentation.

**Background**

  

- *Melanostoma mellinum is a small hoverfly with yellow and black markings, perhaps using this aposematic colourway to loosely mimic wasps and so gain protection from predators *

This sentence is odd for the first sentence of a background. (1) Many hoverflies are assumed to mimic wasps not just *M. mellinum*. (2) The word ‘perhaps’ suggests that this is rather a musing instead of a factual description.

 I would (1) prefer to start describing a trait that stands out for this species in particular and (2) move factual descriptions towards the start of the paragraph and include assumptions towards the end of the paragraph.

  

- *Migration in this species may function to reduce*

“Migration in this species might reduce” would be a better sentence in my opinion.

  

- *This is the first production of a high quality* *M. mellinumgenome and we believe that the sequence described here, generated as part of the* *Darwin Tree of Life project* *, will further aid understanding of the biology and ecology of this hoverfly.*

I encourage the authors to elaborate on this. Do you think your dataset will assist researchers in answering specific research questions regarding the biology and ecology of this species? Do you expect (or even observe) duplication or loss events for certain genes or genes of certain gene families in comparison to other closely related taxa?

 The public availability of new molecular biologicals dataset is in itself valuable, but I would wish for a better description of the project’s rationale.

**Figures**

 Figure 3 should be edited to make it more accessible:

1. I consider my eyes in good condition but found the labels, legends and scale numbers in this figure too small to read. The figure uses a lot of white space. Changing the font sizes might reduce white space and make the plot more appealing to look at.
2. I prefer figures that are self-explanatory. This figure could be labeled more clearly. Specifically: the x-axis is labeled: gc, a more appropriate label would be “GC content”, the y-Axis is labeled: ERR6688428_cov, a more appropriate label would be “Coverage”.
3. It took me a while to understand what was meant by the word ‘phylum’ in the figure description. Firstly, the colors were difficult to distinguish from each other; secondly, the labels in the upper right corner were badly visible; thirdly, the method of using buscogenes taxrule to classify phyla is described in the description of Figure 4. It would be better to mention it in figure 3 as well. Furthermore, I find the term ‘phylum’ inaccurate here. Since the only classes are ‘Arthropoda’ and ‘no-hit’ (and the third class: ‘total’, which is a composite of ‘Arthropoda’+’no-hit’.

I am aware that this plot is automatically generated and is supposed to look similar to the plot in the provided interactive version to which you linked. But I suggest to either polish the presentation of this Figure, or move it to the supplement.

 You might want to consider some of these suggestions for Figure 4 as well. It looks better than Figure 3, but I found the legend describing the ‘phyla’ and also the numbers on the scale too small to read.

 In summary, I think this data does a good job in sufficiently describing the generation of the associated dataset and I approve of this article with minor revisions.

Are sufficient details of methods and materials provided to allow replication by others?

Yes

Is the rationale for creating the dataset(s) clearly described?

Partly

Are the datasets clearly presented in a useable and accessible format?

Yes

Are the protocols appropriate and is the work technically sound?

Yes

Reviewer Expertise:

I am a bioinformatician who has primarily worked with Illumina sequencing data of Arthropods before (primarily Hymenoptera, but I have also worked with sequencing data from hover flies). More recently, my professional interests shifted towards biomedical informatics. I analyze the Illumina sequencing data of tumor biopsies from human patients. I have theoretical, but no practical experience with long-read sequencing data and while I'm experienced in bioinformatics, I cannot evaluate the laboratory protocols used.

I confirm that I have read this submission and believe that I have an appropriate level of expertise to confirm that it is of an acceptable scientific standard.
